# Towards reconciling population nutrition goals and investment policy in Thailand: understanding how investment policy actors defined, framed and prioritised nutrition

**DOI:** 10.1186/s12992-022-00888-4

**Published:** 2022-11-12

**Authors:** Sirinya Phulkerd, Ashley Schram, Jeff Collin, Anne-Marie Thow, Yandisa Ngqangashe, Carmen Huckel Schneider, Sharon Friel

**Affiliations:** 1 Institute for Population and Social Research, Mahidol University, Salaya Campus, 999 Phutthamonthon 4 Road, Phutthamonthon, 73170 Nakhon Pathom, Thailand; 2 Menzies Centre for Health Governance, School of Regulation and Global Governance, Australian National University, Canberra, Australia; 3 Global Health Policy Unit, School of Social & Political Science, University of Edinburgh, Edinburgh, UK; 4 Menzies Centre for Health Policy and Economics, School of Public Health, University of Sydney, Sydney, Australia

**Keywords:** Ultra-processed foods, Discursive power, Problematisation, Investment policy, Population nutrition, Thailand

## Abstract

**Background:**

Government’s investment policy is an important driver of food system activities, which in turn influence consumers practices, dietary consumption patterns and nutrition-related health of populations. While governments globally have committed to developing coherent public policies to advance population nutrition, the objectives of investment policies are seen as being divorced from nutrition and health goals. This study aimed to examine investment policy in Thailand and explore how key actors variously define and frame their objectives in food investment policy, how nutrition issues are represented by the actors, and what discursive effects of the nutrition results were represented within the field of investment in Thailand.

**Methods:**

This study conducted semi-structured interviews with 16 actors (from 23 recruited actors) from government, civil society, academia and industry. A coding framework was developed based on Bacchi’s analytical framework encapsulated in the question “What’s the problem represented to be?” which examines the problem and assumptions underlying a policy. Data coding was first undertaken by a lead researcher and then double-coded and cross-checked by research team. Disagreements were resolved with discussion until consensus was achieved. The interview data were analysed using thematic analysis.

**Results:**

The principal “problem” represented in food investment policy in Thailand was the perceived irrelevance of nutrition to governmental commitments towards increasing productivity and economic growth. Technological innovation in food production and processing such as ultra-processed foods was perceived as a key driver of economic growth. The key assumption underlying this representation was the primacy of a “productivist” policy paradigm, via which the government focuses on industrially driven food and agriculture and expansion to increase productivity and economic growth. This entails that the nutrition needs of Thai people are silenced and remain unacknowledged in investment policy contexts, and also does not take cognisance of the term “nutrition” and its importance to economic growth.

**Conclusion:**

The findings show that nutrition was not perceived as a political priority for the government and other investment actors. Promoting productivity and economic growth were clearly positioned as the primary purposes of investment within the dominant discourse. Nutrition regulation, particularly of UPF, may conflict with current investment policy directions which prioritise development of modern food production and processing. The study suggests that comprehensive policy communication about nutrition and food classification is needed.

## Background

Public sector investment policy [hereafter investment policy] is an important driver of food system activities such as production, processing, procurement and distribution, which in turn influence consumers practices, dietary consumption patterns and nutrition-related health of populations [1]. While governments globally have committed to developing coherent public policies to advance population nutrition [2, 3], the objectives of investment policies are seen as being divorced from nutrition and health goals [4].

Public sector investment policy covers a wide range of government policies, regulations, and legislation that aim to promote and facilitate public and private investments, including via international investment agreements and the provision of incentives for foreign and domestic investments across the food system. Who invests, where they invest, and what they invest in, is an important, but understudied, determinant of the healthfulness of the food supply – including the availability, affordability, and desirability of ultra-processed food (UPF) products [5]. Although investment policies could steer investment across the food system in ways that enhance the healthfulness of the food supply, and in turn improve nutrition outcomes and economic performance [6], investment policies do not currently include nutrition and health in their objectives [4].

A potentially important step in reconciling tensions between these economic and health objectives involves bringing nutrition and health issues associated with the food system to the attention of policy makers in the investment domain, thus helping them to seriouslyconsidersuch issues. This is part of the problem identification process, which is the first step in the agenda setting stage of policy making [7]. This foregrounds the significance of discursive power as a key source of power in the policy system, used by policy actors to influence which problems come to constitute public issues, and thus become subject to government responsibility and action [8]. Thus, an understanding is needed of how policy actors use discursive power to influence investment policies and realted implications for the inclusion of nutrition goals.

This is particularly important in the Southeast Asia region (SEA) where malnutrition remains a significant threat to human and economic development. Not a single country in the region is on course to meet the global adult obesity targets, and 10 of the 11 countries lag behind the target for diabetes [9]. Malnutrition and diet-related non-communicable diseases (NCD) cost governments millions of dollars in socio-economic costs each year [10]. Despite nutrition being part of the “ASEAN Post-2015 Health Development Agenda” [11], and the interlinkages between economic growth and nutrition being increasingly recognised [12], investment policies that could support and advance nutrition outcomes are not informing a comprehensive policy-making approach by the majority of ASEAN governments [10].

Thailand is one of the ASEAN countries that has shown limited progress towards achieving the diet-related NCD targets, and has made no progress towards achieving the obesity targets. Increased consumption of UPF products, a known risk factor for obesity and NCDs [13], was observed among Thai people across all age groups [14]. For example, ready-to-drink tea was the most consumed sugar-sweetened beverages followed by energy drinks, juice drinks and sport drinks, respectively [15]. Although better nutrition and economic progress are both included within government national development goals, as reflected in the 20-year National Strategy (2018–2037) [16], challenges remain in reconciling the economic investment objectives of the strategy with population nutrition objectives.

Despite impressive progress in social and economic development resulting in Thailand moving from being a low middle-income to an upper middle-income country in 2011, economic growth seems to have diminished significantly which reflects stagnation of productivity growth in the past years [17]. A decline in private investment and stagnation of foreign direct investment have been observed. Thailand has recovered slower from COVID-19 than ASEAN peers and that the economic activity during this pandemic was reported below pre-pandemic period [18]. Public debt also reached over 60% of gross domestic product in 2022. The country’s political context since a military-aligned civilian government first seized power in 2014 have undergone continuing political unrest and partisan conflict [17]. These situations expose Thailand’s systemic political instability that is a key risk for Thailand’s economic outlook, and especially for the country’s status as a foreign direct investment destination. This could move interest or priority of policy makers in investment policy away from non-economic issues.To date little research exists that aims to understand the factors influencing the formulation and scope of food investment policy and the implications for nutrition and health outcomes. While the political science literature identifies the involvement of various actors in developing positions for investment policy [19, 20] - policymakers, private sector, civil society groups and technical experts/researchers - there is limited analysis of what issues they consider important, how they frame these issues in policy discourse, and how different actors lobby to advance these issues and their interests. In this paper, therefore, we examine investment policy in Thailand, the problem representations offered by key actors in investment and the discursive effects of these problem representations. We explore how different actors variously define and frame their objectives in food investment policy, how nutrition issues are represented by the actors, and what discursive effects of the nutrition results were represented within the field of investment in Thailand. Differences and commonalities in positions between the different actors are explored. Understanding how nutrition is framed by diverse actors in investment policy is important to help move towards food investment policies for better nutrition in the Thai population. Results from this study can help advance strategies across SEA countries to strengthen the role that investment policy can play in helping to achieve global nutrition targets.

## Methodology

### Study design and participants

We used a qualitative policy analysis involving interviews with policy actors. The study used purposive sampling to ensure that participants were drawn from the most relevant actor sectors: government, technical expert, civil society and industry. Actors involved in decision-making or regulatory roles for food investments in the government-funded system or who have advocated for nutrition/health in investment policies were identified and invited to interview.

A list of relevant actors was initially drawn from secondary data sources, including governmental and nongovernmental websites and documents, and Internet searches. The study supplemented this purposive sample with snowball sampling to identify additional relevant actors. At the end of each interview, participants were asked if they knew any other actors that might be interested in participating in the study. A total of 23 key actors were invited to participate – 11 actors from governmental organisations (GO), three from civil society organisations (CS), four technical experts (TE) and five from the food industry (FI). Sixteen actors agreed to be interviewed, comprising nine representatives from GO (one from health sector and 8 from non-health sector), two from CS, three TE, and two from FI..

- The GO actors are senior-level policy makers in a government department, bureau or division, with direct experience or involvement with policy making in food investment, or other related field;
- The CS actors are senior-level representatives from civil society organisations who have experience related to food investment or similar field;
- The TE actors are university professors, researchers or persons who have specific knowledge or expertise in food investment; and
- The FI actors are senior-level representatives from food companies or organisations that had been working related to food investment.

### Theoretical framework

We examined discursive power using a theoretical framework outlined by Bacchi 2009. Discursive power refers to “the power to influence policies and the political process as such through the shaping of norms and ideas [21],” and “the way an issue is constructed or a problem perceived and how this influences the type of action that is taken [22].” Bacchi’s framework probes the ways that policy issues are positioned through language, specifically: “What is the problem represented to be? (WPR) What are the effects produced by this representation? How are agents or actions constituted in the representation? Who is likely to benefit? What is left unproblematic? [23]” We also considered other perspectives of power such as structural and instrumental power of transnational corporations that are often important in influencing changes in international financial regulation and competition for investment, as well as in the organization of production processes, alongside the exercise of power primarily via lobbying and campaign and party finance activities [24]. The term structural power refers to actors’ ability to set rules, and reward and punish other actors for policy choices, from various sources of power such as finance, production, security and knowledge [25]. The term instrumental power refers to specific mechanisms of control of government policy, such as lobbying and campaign finance.

Previous studies have shown the utility of this approach, providing a systematic way to explore the relationship between discourse and other factors such as power, ideologies and institutions [26–29]. The framework has been used previously to study trade policy and nutrition [22]. Therefore, this approach was considered helpful for guiding a critical analysis of issue framing with respect to investment policy and nutrition in Thailand.

### Data collection

The lead researcher (SP) interviewed participants face to face, using a semi-structured interview guide. Each interview collected information on the participant’s role, interest and vision for food investment, understanding of investment policy systems and impacts of investment on nutrition/health, perceptions of what the “problem” is in supporting nutrition issues in investment spaces, and the underpinning presuppositions, silences, and effects of these problem representations.

Interview guides were written in English, translated into Thai language by SP, and then back-translated to verify fidelity by a professional translator to the original wording. Interviewees were asked for written consent before giving an interview. The interviews were conducted in a quiet place that was convenient and safe for the participant and researcher. The interviews were conducted in the local language (Thai). Interviews were audio-recorded and supported by field notes with the participant’s consent. Interviews lasted on average 60 min and were conducted until no new information was being generated.

### Data analysis

The recorded interviews were transcribed verbatim into Microsoft word documents. Transcripts of the interviews conducted in Thai were translated to English by a specialized translator, and double-checked for accuracy by SP. The transcripts were anonymized. Processed data were entered into Nvivo software version 12. A coding framework was developed by two researchers (SP and AS). Data coding was first undertaken by SP and then double-coded and cross-checked by AS. Disagreements were resolved with discussion until consensus was achieved. The interview data were examined according to the six questions of the WPR framework (Table 1).

**Table 1:** Bacchi questions and data analysis codes

Questions	Analysis codes
1. What’s the “problem” represented to be (in food investment policy)?	Problem representation: definitions, explanations or reasons for food investment policy and nutrition outcome
2. What assumptions underpin this representation of the “problem”?	Assumptions: key concepts and categories for food investment policy and nutrition outcome
3. How has this representation of the “problem” come about?	Genealogy: influential actors, developments, decisions or events in the discourse on food investment policy
4. What is left unproblematic in this “problem” representation? Where are the silences?	Silencing: criticism, gaps or limitations in the discourse on food investment policy for nutrition
5. What effects are produced by this representation of the “problem”?	Effects: perspectives or opinions on alignment of food investment policy towards nutrition and health
6. How and where has this representation of the “problem” been produced, disseminated and defended? How has it been and/or how can it be disrupted and replaced?	Solutions: implications for coherence between food investment policy and nutrition and health outcomes

## Results

### What’s the “problem” represented to be in food investment policy?

In Thailand, public sector investment policies, which aims to promote and facilitate public and private investments, were perceived by government actors from food, agriculture and investment sectors as offering a solution for economic improvement, enabling progress in the transition to a high income country. Technological innovation in food production and processing was perceived as a key driver of economic growth; however, government investment in this sector was thought to be insufficient, and thus it was felt that the private sector must be part of the solution. This was reflected by some government actors in the food and investment sector discussing how they commissioned services in accordance with executive policies. In this context, both Thailand’s 20-year National Strategy and the Thailand 4.0 policy promote and support innovation and higher technologies. Core actions of these policies include supporting “S-curve industries^1^” which include food industry, “Food for the Future” initiative, and “Bio-Circular-Green Economic Model or BCG^2^.” These policies aim for Thailand to become “a developed country with security, prosperity and sustainability.”

*The projects under [name of government agency] aim to transform the country into “Thailand 4.0” which means the use of digital technology and innovation in industry as much as possible. The hope is that these innovations will add significant value to Thai products and production processes. The food industry is one of twelve targeted industries in this initiative [S-curve]. The slogan of the program is “Food for the Future.” There are three target areas in this program: Functional Food, Food Ingredients, and Final Food Product […] These three dimensions of Food for the Future are linked with the master plan for food processing industry that was recently issued by the Ministry of Industry* (GO4).

Although government actors in investment generally welcomed investments that promote nutrition/health, they noted that this target was not their “bottom line”, unlike priorities such as “food innovation” or food production using “modern technology.” One government actor said:

*In the case of the food industry, we look at the process of production of food and beverage products, additives/adornments, etc. One criterion is that the methods used in the production process are modern* (GO2).

The emphasis on modern food production and processing was observed by some actors to be problematic for nutrition. As one technical expert mentioned, improving nutrition requires consumption of minimally processed foods and reduced consumption of highly processed foods such as UPF, due to their association with increased health risk. Balancing this with the economic interests of investment policy makers was perceived as challenging.

*We can see more clearly how over-consumption of UPF could become a health problem. If you process some raw product too much, at some point it isn’t food (nutritious) anymore […] if the processing involves adding large amounts of sugar and sodium, then that could become a problem […] You would have to find a way to link the concept of UPF with health and trade and investment so that policy makers could more easily see the connections.* (TE1).

This expert elaborated further that nutrition/health was only invoked by policy makers in investment as a rhetorical device to promote investment.

*they [investment policy makers] seem to be narrowly considering what is feasible for the economy, or what has the potential for the most cost-benefit advantage for the country. They are not considering the health of the population in these policy decisions. […] Some might use that [nutrition] as an advertising pitch – that it will be good for your health – but that is not the focus of the discussion or decision-making* (TE1).

### What assumptions underlie the representation of the “problem”?

Investment policy discourse in Thailand has its roots in a political and economic context. A major idea evident across the interviews was the influence of a “productivist” policy paradigm which puts the government’s focus on industrially driven food and agriculture and expansion to increase productivity and economic growth. Actors from all sectors acknowledged that this paradigm dominates the food investment sector.

*We are always on the look-out for new markets and countries to trade with. We want Thai farmers and producers to diversify more in order to have alternatives when the price of a certain product falls or there is a glut in the market* (G06).

Another government actor in investment noted that an economic argument would be required for the consideration of UPF in food and investment policy, such as cost-effectiveness of using food processing-based classification system.

*We would like to know in advance how much economic benefit would be derived by a certain technological input [from using food classification system based on processing such as UPF]* (GO4).

One government actor in investment emphasised the status of “modern food production” and “foreign direct food investment” as priorities under the revised Investment Promotion Act No. 4 B.E. 2560 (2017) and an investment law named “Competitiveness Enhancement Act”, to create new BOI benefits, which include an increase in technology transfer to the host country.

*This was Thailand’s way of shifting emphasis to upgrading the technological infrastructure and potential of the country across the board. The sectors of interest included biotechnology, nano-technology, advanced-material technology, and digital technology. Any investor which offered these technologies plus a partnership with the relevant Thai educational institutions (to receive technology transfer) then that investor would immediately receive 10-year tax exemption. If the enterprise had other special R&D features and areas of excellence, then the duration of exemption could be further increased* (GO2).

Concerns were also raised by actors from civil society and technical experts, where it was felt that the dominant “productivist” paradigm could undermine the extent to which the government is held to account for nutrition and health outcomes. This was reflected in the prominence of business organisations by the actors.

*The fact that the government hesitated on withdrawing [from joining the CPTPP due to concerns of Thai farmers and civil society that this agreement prevents them from saving and reusing seeds containing patented plant material] means that there is a counterbalance between the social sector against the Federation of Thai Industries and the Thai Chamber of Commerce. Therefore, it is possible to say as well that, supposedly if there is an issue concerning the [public health] measures affecting the investors on processed food, essentially these two organizations will be relevant* (CS2).

Government actors from food and commerce sectors mentioned the international trade and investment regime as a constraining force that underpinned the problem representation in food investment policy. Government actors perceived that compliance with the Codex Alimentarius, and other obligations created by international trade and investment agreements, allow priority for ensuring food safety standards, but largely are in place to facilitate the flow of global trade to support the country’s food supply chain over investment in healthy diets.

*we are bound by these trade agreements [on agricultural products]. So the government can’t deviate too much from those guidelines and standards. It’s not just the Ministry of Commerce acting alone here. […] We have to see if the terms of Codex are complied with […] There are the Technical Barriers to Trade of the WTO that Thailand has to comply with. The purpose is to prevent trade partners from applying undue pressure on each other* (GO5).

One government actor in investment mentioned that they are looking at the use of digital technology and innovation in the industry in order to add significant value to Thai products and production processes. The food industry is one of their ten targeted industries. However, when it comes to health, they did not count health as an industry, and as such nutrition did not seem relevant to their work.

*We don’t view health as an industry. Health is one component in the development of the “Smart City.” That concept involves social indicators of improvement. But that area is not my forte, so I am not sure where nutrition would fit in. The focus is more on lifestyles and access to safe food* (GO4).

Despite these acknowledgements, some government and commercial actors believed that results from modern food production and processing are critical to food security and nutrition.

*Processing food is an important strategy to achieve food security* (GO4).

### How has this representation of the “problem” come about?

The revised Investment Promotion Act No. 4 B.E. 2560 (2017) was identified as posing a barrier to the Board of Investment of Thailand (BOI) being able to prioritise nutrition within investment policy. This reflects the Act’s emphasis on increasing technology-based industries such as processed food and agro industries.

*after the revised law was passed, there was a shift in emphasis from activity-based to technology-based enterprise. Thus, the 8-year limit for tax exemption was maintained for activity-based enterprise, while the 13-year extended limit was applied to technology-based enterprise* (GO2).

The BOI is perceived by participants from all sectors to be the most influential actor. The processes used by industry to influence the BOI were identified as shaping the problem representations. One government actor in investment reflected on the “public and private platform” created for holding a regular dialogue between public and private sector investment actors to discuss or agree on measures related to investment policy issues. The BOI also holds power of unilateral action and with this power it lobbies for bringing in more resources such as expertise.

*[BOI] also look at development of food production technology in agro-industry and hubs like the Food Innopolis [a global food innovation hub at the Thailand Science Park]. […] If there are measures which the BOI can do directly to improve the situation, then the BOI can act unilaterally […] For example, in the past, some have made the case that Thailand lacks experts in certain fields that are critical for the country’s development (e.g., aviation). In that situation, the BOI will consult with the relevant agency/organization and, if it is true, then we will lobby for modifications in the procedures/regulations to make it easier to bring in that expertise in a systematic way* (GO2).

This was consistent with the interview given by one commercial actor emphasising “transparent” public and private sector dialogue to help facilitate investment for hi-tech industry.

*Talking about investment, we would tell all our counterparts that investment was the purview of the BOI. The BOI did not force an entity to be part of the Federation of Thai Industries or Thai Chamber of Commerce, but they did have stipulations on the nature of the investment. Then, there came a time when the emphasis was on high-end technology, but hi-tech can be interpreted in multiple ways. […] This is the direct responsibility of the BOI. They want to do it neutrally, without political interference* (FI1).

By contrast, engagement of health actors in investment policy discussions was described by one technical expert as being mainly “by invitation” only, with attention to health restricted to “healthcare and health services” policy such as Thailand’s medical hub policy and long-term care facilities and nursing homes.

*The first time [I was involved] there was an issue related to FDI [Foreign Direct Investment] in the hospital sector. The issue concerned taxation rights of private hospitals. […] At that time, there was discussion about how this should not be a commercially-led initiative. […] My second involvement with the BOI was two or three years ago. That time the issue was investment in health services outside the hospital setting* (TE1).

### What is left unproblematic in this “problem” representation?

Despite some recognition of the case for aligning investment policies with public health benefits, these policies do not sufficiently take into account the nutrition implications of government investment to enhance future economic and social benefits. Food investment policy tends to be silent on nutrition issues, reinforcing the perception that interventions should focus on economic and technological aspects. Not only does the nutrition of Thai people remain unacknowledged in such policies, these do not take cognisance of the term “nutrition” and its importance to economic growth. Most government actors in investment are not familiar with food and nutrition policy objectives and as such do not prioritise it as a driver of economic wellbeing.

*I don’t think they are considering the [food and nutrition] issue from a broad enough perspective. They [policy makers] are not considering the diversity of the context and the dynamic way the challenge is evolving. Instead, they seem to be narrowly considering what is feasible for the economy, or what has the potential for the most cost-benefit advantage for the country. They are not considering the health of the population in these policy decisions* (TE1).

Perceptions on approaches to food classification constitute another gap in the representations. Although some government and industry actors and technical experts generally welcomed the idea of classifying foods as “healthy” and “unhealthy” based on level of processing to promote better nutrition, many pointed to unclear definitions within this food classification system, limited information on its health related impact, and challenges in promoting healthy eating through a return to traditional (or less processed) food diet. Thus this can affect prospects for regulating food investment for nutrition.

*I don’t think that classifying products as UPF would change the policy climate. That’s because people don’t yet have an intuitive understanding of UPF. But in the academic field or health field, we can see more clearly how over-consumption of UPF could become a health problem* (TE1).

*there has to be strong scientific data to back up a position. However, if the evidence is ambiguous, then it is hard to find a middle ground. This is especially true for the health impacts of certain products [such as UPF]. Diets of different populations around the world have evolved over a period of thousands of generations, and the human body has adapted to the local diet out of natural selection. Thus, what is nutritious for one tribe may be toxic for another tribe. However, with globalization, diets are merging. People who used to subsist on a diet of, say, cactus root, now eat burgers and fries as a meal. Once a people transitions too far away from their traditional diets, it is hard to go back* (FI1).

One commercial actor argued for allowing people to enjoy unhealthy foods on occasion and letting them eat in balance and moderation.

*once in a while, it is OK to eat junk food or other food that doesn’t have much nutritional value. We can do that just as a diversion, for fun. But, the average meal needs to be a balanced diet* (FI2).

Concern was also raised from one government actor in commerce where it was felt that UPF interests may clash with current investment policy directions.

*Insects are made into sausages. They are ultra-processed foods? […] Thailand should sell anything that adds value to it. In order to add value, it requires technology, requires industrial production, and then, and of course ah, that food item has to be processed more or less as you said. It could have gone to Group 4, ultra, or just a little bit of processing, from shrimp to breaded shrimp, teriyaki shrimp, something like this, which is what if you think that it affects [our investment policy] or not, I think it affects* (GO8).

Opposition to UPF was particularly prevalent among commercial actors. It was felt that use of this food classification system would “stigmatise” their products as harmful. These actors argued that their business is supporting Thais’ modern lifestyle and food security.

*I am concerned about stigmatizing certain foods arbitrarily. The term ‘processed food’ is acquiring a bad reputation. Some Thais are even refusing to eat any processed food. UPF is seen as the worst in this category. […] So, this is a case of an UPF that is indispensable in a modern society where women are expected to work outside the home. If the term ‘UPF’ acquires a negative connotation in the mind of the consumer, that could do damage when certain UPF are essential. […] if we also characterize UPF as using innovative processes to improve food security through, for example, extending shelf life, or making a fixed amount of food more nutritious, or tailoring the food to the age group of the consumer, that would help improve the general public’s understanding of UPF* (FI2).

Another limitation for the investment discourse is that the existing mechanisms for consultation do not drive diverse stakeholder engagement. Actors representing nutrition and health interests were generally excluded from broader discussions, and thus ideas about nutrition and health are in effect silenced within the investment policy arena. Despite acknowledgement of government to engage multisectoral actors in policy discussions, existing platforms appear to be appear ro be privileging engagement between government and the commercial sector. Civil society actors felt underrepresented in these processes. They were of the view that existing processes limited their opportunities to be involved or provide input into investment policy. Despite being invited to participate, the government response could be perceived as tokenistic, not taking their input seriously or that their involvement was a government afterthought. However, this could be improved with public pressure.

*they [government] probably wouldn’t dare to invite us. If they do, how they listen to what we said is another story. Later they saw that if they didn’t listen to us, the point we were talking about, would be an issue that people echoed up, it’s really a problem* (CS1).

The health sector did not appear to be recognised as a key part of the multisectoral coordination platform led by the investment sector:

*The Board has representatives from the economic, finance, industrial, and commerce sectors of the government, as well as the President of the Federation of Thai Industries, and the Chairman of the Chamber of Commerce. Therefore, any policy that comes out is considered to be approved by both the public and private sectors* (GO2).

## Discussion

This study presents an analysis of the power of different actors in Thai food investment policy through Bacchi’s theoretical lens of examining what is the policy problem represented to be. To our knowledge, this is the first study to subject this policy domain to an analysis relating to the problematistion of nutrition in food investment in Southeast Asian countries.

The study scrutinized the framing of problems and solutions, exploring a key set of influences in investment discourse and governance for nutrition and health through domestic consumption in Thailand in a manageable step-by-step procedure using the WPR approach.

Nutrition was problematised as an issue as being irrelevant within investment policy. It was not a political priority for the Thai Government nor among investment actors. Government policies and actions related to investment focused primarily on moving the country towards becoming an economically developed country, including through an emphasis on promoting technological innovation in food productionand processing [30]. Participants acknowledged the importance of nutrition issues such as food security in their rhetoric but not in their policy solutions, thereby helping to marginalize nutrition as irrelevant for investment actors. The representations of irrelevance of nutrition stand in contrast to the representations made by public health, academic and civil socity community which depict nutrition as an issue influenced by agriculture and food systems investments that enable the availability, access, and affordability of healthy diets [1, 4, 20].

Productivity and economic growth were positioned as the primary purpose of investment within the dominant discourse. This application of a productivist paradigm to investment policy strongly emphasises the objectives of promoting “modern food production” and “foreign direct food investment” as capable of adding significant value to Thai products and production processes and thus increase productivity and accelerate country economic growth. This paradigm, which is strongly institutionalised in Thailand, aligns deeply with a neoliberal ideology characterised by primacy of market-oriented policies with a minimal role for government intervention or industry regulation [31]. This market ideology can lead to the replacement of traditional agriculture and healthy diets with high-energy dense and nutrient-poor foods such as ultra-processed foods which are associated with increased risk of NCDs [32, 33]. This may be an important barrier to promoting consideration of population nutrition goals in investment policy. This replacement also impacts social aspects such as reduced numbers of farming communities, rural-to-urban migration, low-wage employment and greater economic inequity [31], carrying high costs to both population health and societal wellbeing. Despite references to nutrition through food security by non-health participants, they were predominantly rhetorical. Without translation to more substantive politicial commitments, this rhetoric is limited in its capacity to achieve nutrition goals [34].

The economic benefits of investing in nutrition have been clearly demonstrated. Poor nutrition affects economic productivity both directly – such as through low work performance in undernourished worker [35], and indirectly such as via effects on individual cognitive performance [36] and academic achievement [37]. Although nutrition was acknowledged rhetorically by non-health actors from government and industry, it was argued that nutrition regulation, particularly of UPF may conflict with the current investment policy directions which promote modern food production and processing. Some actors also saw UPF as opportunity to extend its shelf-life, promote food safety, and improve food security in Thailand. UPF was seen to bring convenience which was perceived as integrating with features of every life in a modern society. This has been observed elsewhere, and has been described as paying “lip service” to denote contexts where actors acknowledge importance of health, yet actions tend to be neither driven by nor target health-reated goals [38, 39]. To overcome opposition to regulating UPF, particularly by commercial actors, the government may consider to use financial incentives and planning regulations to business to produce, market and promote nutritious foods. The government can promote unprocessed and minimally processed food products and meals in the markets, through whole food reformulation or food innovations alongside with agricultural and economic incentives such as non-UPF subsidies. Investment funds and technical support can be provided that is focused on start-ups and small-and medium-sized food processing business. The government can apply food-based classification system in combination with Thailand’s nutrient profile model in order to take account of both nutritional components and food processing.

This study acknowledges some limitations. The study sought to analyse how population nutrition goals are represented in investment policy. Findings do not reflect the extent to which proposed actions have been implemented or evaluated. The study focus was on actor interviews as an indicator of governments’ commitment to act on population nutrition in investment space. Analysis of these representations from other sources of information such as policy documents and news media may be useful to gain a more indepth understanding of the representations from public and politicial opinions.

### Policy implications

The identified silences and absences, as well as presence of nutrition issues, point to potential areas for food investment policy development and change, and encourage actors to think differently about how important nutrition objectives could be understood in investment policies and what the solution to the problem should be. The study findings suggest a number of implications. Improvements are needed in institutionalized processes within government to facilitate cross-sector problem-solving. This cross-sectoral collaboration can be supported through a mixture of “hard and soft governance” mechanisms, ranging from statutory regulation to persuasion and incentives. For example, food innovation can be encouraged as a means of promoting “whole food reformulation - or development of less processed alternatives [40],” and agricultural and economic incentives such as subsidies on unprocessed and minimally processed foods can be offered to encourage such action by industry. This would not only make their products more affordable for Thai consumers and promote development of less processed products, but also maintain company profits and the country’s economy.

The study also suggests a need for comprehensive communication about nutrition and food classification such as UPF, and particularly for developing a coordinative and communicative discourse among policy makers. A clear understanding and workable definition of healthy and unhealthy foods, and of the particular issues regarding UPF, is fundamental in order to effectively promote nutrition outcomes in investment policy. Knowledge on these issues should be packaged in a way that is persuasive to policy makers, including as policy briefs [41], and be communicated both internally and externally, such as at meetings and hearings for and with citizens, government actors, businesses, interest groups and educational institutes.

In addition, participants cited disruptions to the nutrition agenda due to the influence of industry actors. Developing “tracking systems” to monitor industry lobbying of the government could help and greater accountability in diversity of stakeholders [42]. This requires whole-of-society consultation that involves third parties such as civil society within governing process [43]. Such an approach can enhance government legitimacy, generate additional social capital, and ensure local needs are reflected, and gain trust and access to valuable resources from the third parties.

## Conclusion

This study highlights a number of implications for addressing population nutrition goals in Thailand’s investment policy. The study suggests that a mixture of measures ranging from regulation, to persuasion and incentives can be useful for facilitating this cross-sector problem-solving and encouraging joint action of health and investment sectors. The study also suggests a need for comprehensive communication about nutrition and food classification such as UPF to policy makers through effective forms of policy communication such as policy brief. Monitoring systems of industry lobbying of the government is necessary to ensure better public accountability for nutrition.

## Data Availability

The datasets generated and/or analyzed during the current study are not publicly available to ensure privacy of interviewees. Access to anonymized interview transcripts and archival data is available from the corresponding author on reasonable request.

## Abbreviations

ASEAN: The Association of Southeast Asian Nations
BOI: The Thailand Board of Investment
CS: Civil society organisation
GO: Government organisation
FI: Food industry
NCDs: Non-communicable diseases
SEA: Southeast Asia Region
TE: Technical expert
UPF: Ultra-processed foods
WPR: What’s the problem represented to be

## Footnotes

1 The ten S-curve industries have been targeted as the best industries to promote country investment under the Thailand 4.0 industrial development concept. They include industries in food processing, agricultural and biotechnology, smart electronics, affluent medical and wellness tourism, next-generation automotive, biofuels and biochemical, digital economy, medical hub, automation and robotics, and aviation and logistics.

2 BCG is a new model which underpins Thailand 4.0 policy to drive the economic and social development of the country through the promotion of the bioeconomy (B), circular economy (C) and green economy (G).

## References

[1] FAO (2020). Nutrition-sensitive investments in agriculture and food systems – Budget analysis guidance note.

[2] FAO/WHO, editor Rome Declaration on Nutrition. Second International Conference on Nutrition; 2014; Rome: Food and Agriculture Organization of the United Nations and the World Health Organization.

[3] United Nations. Secretary-General’s chair summary and statement of action on the UN Food Systems Summit New York. United Nations; 2021 [Available from: https://www.un.org/en/food-systems-summit/news/making-food-systems-work-people-planet-and-prosperity.

[4] United Nations System Standing Committee on Nutrition (2016). Investments for healthy food systems. A framework analysis and review of evidence on food system investments for improving nutrition.

[5] FAO I, UNICEF, WFP and WHO (2021). The state of food security and nutrition in the world 2021. Transforming food systems for food security, improved nutrition and affordable healthy diets for all.

[6] WHO Commission on Macroeconomics and Health (2001). Macroeconomics and health: investing in health for economic development/report of the Commission on Macroeconomics and Health.

[7] Sabatier PA (2007). Theories of the policy process.

[8] Schattschneider EE (1975). The semi-sovereign people: a realist’s view of democracy in America.

[9] South-eastern Asia. The burden of malnutrition at a glance [Internet]. Development Initiatives. n.d. Available from: https://globalnutritionreport.org/resources/nutrition-profiles/asia/south-eastern-asia/.

[10] ASEAN/UNICEF/WHO (2016). Regional Report on Nutrition Security in ASEAN.

[11] The Association of Southeast Asian Nations (2018). ASEAN Post-2015 Health Development Agenda.

[12] Ghosh S (2018). India: nutrition intake and economic growth, a causality analysis. Dev Stud Res.

[13] Chen X, Zhang Z, Yang H, Qiu P, Wang H, Wang F (2020). Consumption of ultra-processed foods and health outcomes: a systematic review of epidemiological studies. Nutr J.

[14] National Health Examination Survey Office (2011). National food consumption survey in 2008-9.

[15] Passport global market information database [Internet]. 2021 [cited November 11, 2021].

[16] Office of the National Economic and Social Development Council. Thailand’s 20-year National Strategy Bangkok: Office of the National Economic and Social Development Council; n.d. [Available from: http://nscr.nesdc.go.th/%E0%B8%A2%E0%B8%B8%E0%B8%97%E0%B8%98%E0%B8%A8%E0%B8%B2%E0%B8%AA%E0%B8%95%E0%B8%A3%E0%B9%8C%E0%B8%8A%E0%B8%B2%E0%B8%95%E0%B8%B4/.

[17] World Bank Group (2022). Country private sector diagnostic. Creating markets in thailand. Rebooting productivity for resilient growth.

[18] World Bank Group (2022). Thailand economic monitor: Building back greener: The circular economy.

[19] Behrman JN (1974). Actors and factors in policy decisions on foreign direct investment. World Dev.

[20] Garton K, Thow AM, Swinburn B (2021). International trade and investment agreements as barriers to food environment regulation for public health nutrition: A realist review. Int J Health Policy Manage..

[21] Fuchs D (2005). Commanding heights? The strength and fragility of business power in global politics. Millennium.

[22] Friel S, Ponnamperuma S, Schram A, Gleeson D, Kay A, Thow A-M (2016). Shaping the discourse: What has the food industry been lobbying for in the Trans Pacific Partnership trade agreement and what are the implications for dietary health?. Crit Public Health.

[23] Bacchi C (2009). Analysing policy: what’s the problem represented to be?.

[24] Fuchs D (2007). Business power in global governance.

[25] Clapp J, Fuchs D, Clapp J, Fuchs D (2009). Agrifood corporations, global governance, and sustainability: A framework for analysis. Corporate power in global agrifood governance.

[26] Van Aswegen J, Hyatt D, Goodley D (2019). A critical discourse problematization framework for (disability) policy analysis. Qualitative Res J.

[27] Regmi KD (2019). Critical policy sociology: key underlying assumptions and their implications for educational policy research. Int J Res Method Educ.

[28] Alexander SA, Coveney J (2013). A critical discourse analysis of Canadian and Australian public health recommendations promoting physical activity to children. Health Sociol Rev.

[29] Lappalainen S, Nylund M, Rosvall P-Å (2018). Imagining societies through discourses on educational equality: A cross-cultural analysis of Finnish and Swedish upper secondary curricula from 1970 to the 2010s. Eur Educational Res J.

[30] Anuroj B. Thailand 4.0 – a new value-based economy. Bangkok: Thailand Board of Investment; n.d.

[31] Nestle M (2019). How neoliberalism ruins traditional diets and health. The Lancet Diabetes & Endocrinology.

[32] Elizabeth L, Machado P, Zinöcker M, Baker P, Lawrence M. Ultra-processed foods and health outcomes: a narrative review. Nutrients. 2020;12(7):1–33. 10.3390/nu12071955 PMC7399967 32630022

[33] Juul F, Vaidean G, Parekh N (2021). Ultra-processed foods and cardiovascular diseases: potential mechanisms of action. Advances in nutrition (Bethesda. Md).

[34] Baker P, Hawkes C, Wingrove K, Demaio AR, Parkhurst J, Thow AM (2018). What drives political commitment for nutrition? A review and framework synthesis to inform the United Nations Decade of Action on Nutrition. BMJ Global Health.

[35] Ross J, Chen CM, He W, Fu G, Wang YY, Fu ZY (2003). Effects of malnutrition on economic productivity in China as estimated by PROFILES. Biomed Environ Sci: BES.

[36] Wright RS, Gerassimakis C, Bygrave D, Waldstein SR (2017). Dietary factors and cognitive function in poor urban settings. Curr Nutr Rep.

[37] Naveed S, Lakka T, Haapala EA (2020). An overview on the associations between health behaviors and brain health in children and adolescents with special reference to diet quality. Int J Environ Res Public Health.

[38] Mukuru M, Kiwanuka SN, Gilson L, Shung-King M, Ssengooba F (2021). "The actor is policy”: application of elite theory to explore actors’ interests and power underlying maternal health policies in Uganda, 2000–2015. Int J Health Policy Manag.

[39] Junquera B. Paying lip service to health: an analysis of health in climate change mitigation policies in Spain. J Climate Change Health. 2022;6:100128.

[40] Adams J, Hofman K, Moubarac J-C, Thow AM (2020). Public health response to ultra-processed food and drinks. BMJ.

[41] McBride T, Coburn A, Mackinney C, Mueller K, Slifkin R, Wakefield M (2008). Bridging health research and policy: effective dissemination strategies. J public health Manage practice: JPHMP.

[42] Swinburn B, Kraak V, Rutter H, Vandevijvere S, Lobstein T, Sacks G (2015). Strengthening of accountability systems to create healthy food environments and reduce global obesity. The Lancet.

[43] Bell S, Hindmoor A (2009). The governance of public affairs. J Public Affairs.

